# Road traffic flow prediction based on dynamic spatiotemporal graph attention network

**DOI:** 10.1038/s41598-023-41932-6

**Published:** 2023-09-07

**Authors:** Yuguang Chen, Jintao Huang, Hongbin Xu, Jincheng Guo, Linyong Su

**Affiliations:** Faculty of Transportation Engineering, Kunming University of Science and Technology, Kunming, 650500 China

**Keywords:** Mathematics and computing, Scientific data, Civil engineering

## Abstract

To improve the prediction accuracy of traffic flow under the influence of nearby time traffic flow disturbance, a dynamic spatiotemporal graph attention network traffic flow prediction model based on the attention mechanism was proposed. Considering the macroscopic periodic characteristics of traffic flow, the spatiotemporal features are extracted by constructing spatiotemporal blocks with an adjacent period, daily period, and weekly period respectively. The spatiotemporal block is mainly composed of a two-layer graph attention network and a gated recurrent unit to capture the hidden features of space and time. In space, based on considering adjacent road segments, the Pearson correlation coefficient is used to capture the hidden correlation characteristics between non-adjacent road segments according to a certain time step. In terms of time, due to the random disturbance of traffic flow at the micro level, the attention mechanism is introduced to use the adjacent time as the query matrix to weight the output characteristics of daily cycle and weekly cycle, and the three are connected in series to output the prediction results through the linear layer. Finally, the experimental results on the public data sets show that the proposed model is superior to the six baseline models.

## Introduction

As an important basis for traffic planning, traffic management, and control, traffic flow prediction is one of the most critical problems in intelligent transportation system research. With the widespread deployment of intelligent data acquisition terminals such as Radar and GPS devices, a large amount of data reflecting the state of the road network has been accumulated, and massive traffic data provides a good foundation for traffic flow prediction. Therefore, how to improve prediction efficiency and accuracy by optimizing the prediction method has become a key issue.

Traffic flow prediction is mainly to predict the traffic flow parameters of a certain region in a future period based on historical traffic data^1^. For this reason, the early effort to predict mostly predict based on time series prediction models, such as Xingwei et al.^2^, which compared ARIMA and LSTM for traffic flow prediction. Kumar et al.^3^ proposed a short-term traffic flow prediction method based on SARIMA to overcome the availability problem. Since the traffic flow is affected by many factors, the general parameter method cannot adapt to the complex random characteristics of traffic flow. Therefore, some machine learning methods have been applied to traffic flow prediction. For example, Castro-Neto et al.^4^ proposed an online regression support vector machine model (OL-SVR) for highways under typical and atypical conditions, and the research results show that the model has better performance under atypical traffic conditions. Wang et al.^5^ assume that in an expected interval, the traffic flow is related to the traffic flow in previous intervals, and propose a new Bayesian method for traffic flow prediction based on the grey relational analysis method of entropy.

With the acquisition of high-fine-grained spatiotemporal data of road traffic flow becoming more and more convenient, traditional machine learning methods perform poorly in dealing with high-dimensional and complex spatiotemporal data. Deep learning has certain advantages in mining high-dimensional features of data. Many deep learning models such as Convolutional Neural Networks (CNN)^6–8^, Recurrent Neural Networks (RNN)^9,10^, and Generative Adversarial Networks (GAN)^11–13^ have powerful feature processing capabilities, so they have good prediction effects in traffic prediction tasks. In addition, variants of recurrent neural networks such as Long Short-Term Memory (LSTM)^9,14^ and Gated Recurrent Unit (GRU)^15,16^ are also widely used due to their powerful time series data processing ability. To better extract data features and give full play to the advantages of each model, researchers enhance the performance of the model by stacking different networks. For example, Ma et al.^17^ proposed to use Convolutional Neural Network (CNN) to extract daytime and intra-day traffic flow patterns. Then, the extracted features were input into the Long Short-Term Memory (LSTM) unit to learn the intra-day time evolution process of traffic flow. This method captures the temporal correlation of traffic flow well, but ignores the spatial correlation of traffic flow. Fang et al.^18^ proposed a long short-term memory network attention mechanism considering the long-term dependence of traffic flow evolution. Experiments have proved the effectiveness of the model in capturing subtle fluctuations of traffic flow, but there are still shortcomings in capturing long-term dependence. Attention mechanism^19^ helps the network model to focus on key information and ignore small fluctuations. Jian-xi et al.^20^ used multi-scale convolution kernel to capture multi-factor nonlinear correlation and spatial correlation of road network nodes, extracted time features through gated recurrent unit, and proposed a multi-periodic component spatiotemporal neural network model, which considered the periodic characteristics of traffic flow. Wang et al.^21^ considered road traffic flow data and weather conditions, introduced an attention mechanism to enable the model to focus on learning more important data features, and proposed a short-term traffic flow prediction model based on the attention mechanism of 1DCNN-LSTM network. Experiments show that the introduction of multiple traffic environment parameters plays an important role in improving the performance of the model. Jia et al.^22^ proposed a grouping residual network model based on self-attention mechanism to predict urban traffic flow, and the experiment shows that the prediction effect of the model is good. Using CNN can extract spatial relationships in Euclidean space, however, there are certain limitations in feature extraction in non-Euclidean space. In recent years, graph neural networks have been widely used in spatial information extraction of road networks due to their powerful performance in capturing the structural features of graph networks^23^. Guo et al.^24^ proposed a new Attention-based Spatiotemporal graph Convolutional Network (ASTGCN) model to solve the traffic flow prediction problem based on the three time characteristics of traffic flow, namely recent, daily and weekly cycle dependencies. Experiments show that the model can well capture the spatiotemporal correlation characteristics of traffic flow, but it only considers the direct links between road segments and ignores the indirect links, that is, ignores the correlation of upstream road segments. Li et al.^25^ effectively learned the hidden spatiotemporal dependencies on the basis of the temporal graph and proposed a spatiotemporal fusion graph neural network (STFGNN) for traffic flow prediction. Wang et al.^26^ constructed an attention-based Spatiotemporal Graph attention network (ASTGAT) model by using an attention mechanism, dilated gated convolution, and graph attention network. The results proves that the model can effectively extract in-depth spatiotemporal information, and the prediction effect is significant excelled other algorithms.

The existing research has achieved abundant achievements, but there are still the following shortcomings: (1) The traffic flow prediction task is simply regarded as a time series prediction task, ignoring the spatial correlation characteristics, and cannot make good use of the traffic information of upstream and downstream traffic flows; (2)Simply defining the neighbor matrix based on the geographical location distance of nodes cannot describe the spatial correlation of non-adjacent sections, and some potential similar traffic flows are ignored; (3) Feature extraction of time series is mostly based on the data of the same location at different times, and the impact research on the current prediction period is insufficient, neglecting the feature extraction under the influence of unexpected traffic incidents; (4) GCN is suitable for processing static undirected graphs, while traffic flow is time-varying, and it is usually difficult to deal with a dynamic adjacency matrix.

The main contributions of this paper are as follows:

1. A traffic flow prediction model based on Dynamic Spatiotemporal Graph Attention Network (DSTGAT) is proposed. Experiments show that the prediction accuracy of the model is high.
2. Considering the indirect links of non-adjacent sections, the Pearson correlation coefficient is used to extract the correlation features of the traffic sequence of non-adjacent nodes.
3. DSTGAT is used to effectively learn the hidden spatiotemporal relationship of traffic flow.
4. The attention mechanism is introduced to enhance the performance of the model to capture the impact of microscopic traffic incidents according to the traffic flow fluctuation characteristics in the adjacent period, and the prediction effect of the model is improved.

The rest of this paper is organized as follows: “Matrix construction and problem description” section mainly constructs the adjacency matrix and introduces the problem definition of traffic flow prediction. “Methods” section describes the model structure and model details in detail. “Experiment” section is mainly for experimental verification, which mainly includes introducing data sets, data preprocessing, and parameter setting, then compares the evaluation indicators with the baseline models, and analyzes the results. “Discussion and conclusions” section is the conclusion and outlook of the paper.

## Matrix construction and problem description

Traffic flow prediction is the process of forecasting traffic conditions for a future period based on historical traffic information. We define a road network graph, denoted as a graph $G = (V,E{,}A)$, to represent the topological structure of the road network. The set of road nodes is represented by $V \in R^{N}$, where *N* represents the number of nodes. $E \in R^{N \times N}$ represents the set of edges, and the adjacency matrix $A \in R^{N \times N}$ primarily describes the connectivity between road segments.

1 $$A(i,j) = \left\{ {\begin{array}{*{20}l} {1,} \hfill & { < i,j > \in E{\kern 1pt} \;or\;(i,j) \in E} \hfill \\ {0,} \hfill & {{\text{otherwise}}} \hfill \\ \end{array} } \right.$$

where $< i,j > \in E{\kern 1pt} \;or\;(i,j)$ represents the node pair composed of node *i* and node *j*.

In the road network, it is not necessarily the adjacent road section that is associated with the target road section. At the same time step, different road sections show similar flow characteristics. Considering the linkage characteristics of traffic flow, this paper uses the Pearson correlation coefficient of the same time step between different sections to explore the spatial correlation of non-adjacent sections. Due to the excessive connection relationship and more calculation parameters, we define that when the Pearson correlation coefficient of two road segments is greater than the threshold, there is an implicit connection relationship between two road segments. In this paper, the threshold is set to 0.9 according to the data experiment, and the spatiotemporal adjacency matrix fusing the implicit connection relationship can be expressed as shown in Eq. (2).

2 $$A_{p} (i,j) = \left\{ {\begin{array}{*{20}l} {1,} \hfill & { < i,j > \in E{\kern 1pt} \;or\;(i,j) \in E|Pearson \ge 0.9} \hfill \\ {0,} \hfill & {{\text{otherwise}}} \hfill \\ \end{array} } \right.$$

The feature matrix $Q \in R^{NF}$ is defined, and the traffic information on the road network is taken as the attribute feature of the network node $Q \in R^{NF}$, where F represents the number of attribute features. Therefore, the traffic flow prediction problem can be expressed as the mapping function *f* predicts the flow at time T in the future by using the direct and indirect association relationship $A_{p} (i,j)$ of road sections and the flow characteristics Q, as shown in Eq. (3).

3 $$Q_{t + T} = f(A_{p} (i,j);(Q^{R} ,Q^{D} ,Q^{W} ))$$

where $Q_{t + T}$ represents the set of road section flow $Q_{t + T} = \left[ {Q_{t + 1} ,Q_{t + 2} , \cdots ,Q_{t + T} } \right]$ predicted at time T in the future of traffic flow. n denotes the length of historical time series; $Q^{R} ,Q^{D} ,Q^{W}$ denotes adjacent time, daily cycle time, and weekly cycle time, respectively.

## Methods

### Architecture of DSTGAT

**Figure 1:**
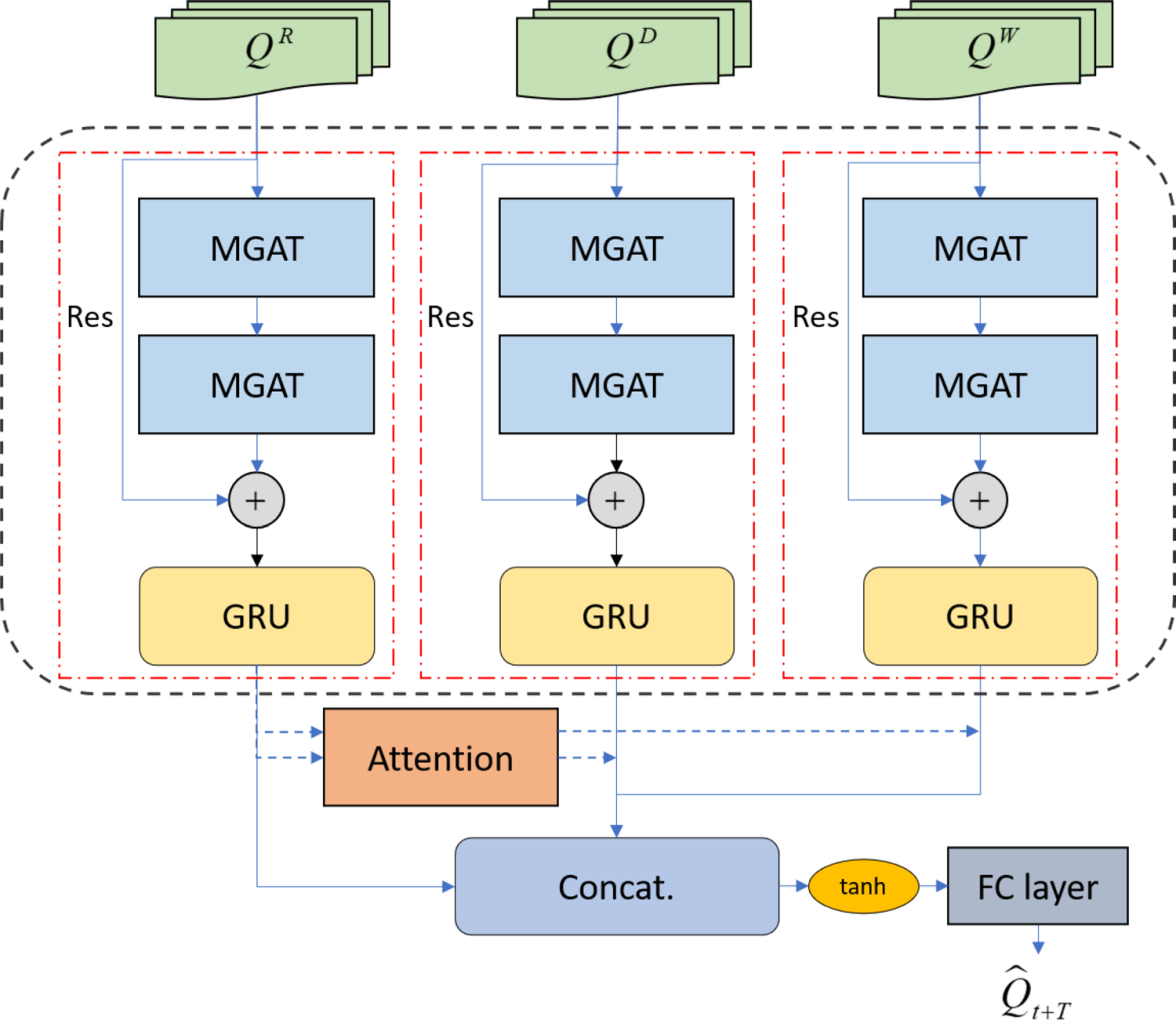
DSTGAT model framework. MGAT, Multi-head graph attention network; Res, Residual network; FC layer, Fully connected layer.

The architecture of the DSTGAT model proposed in this paper is shown in Fig. 1. The framework is composed of three interconnecting space–time blocks. Considering the periodic characteristics of traffic flow, this paper trains the model according to the traffic flow data of three different cycles: adjacency, daily, and weekly cycle time. Each space–time block is composed of two graph attention networks and a gated recurrent unit, which are used to extract the spatial and temporal characteristics of road traffic flow respectively, while adding residual connections to prevent the gradient from disappearing. Then, with the traffic flow data of adjacent time as the main reference, the attention mechanism is used to assign weights to the daily and weekly cycle times output. Finally, the output results of the three components are fused with features, and the predicted value is output after the linear layer. The main model framework of the paper is shown in Fig. 1.

### Spatial feature extraction

**Figure 2:**
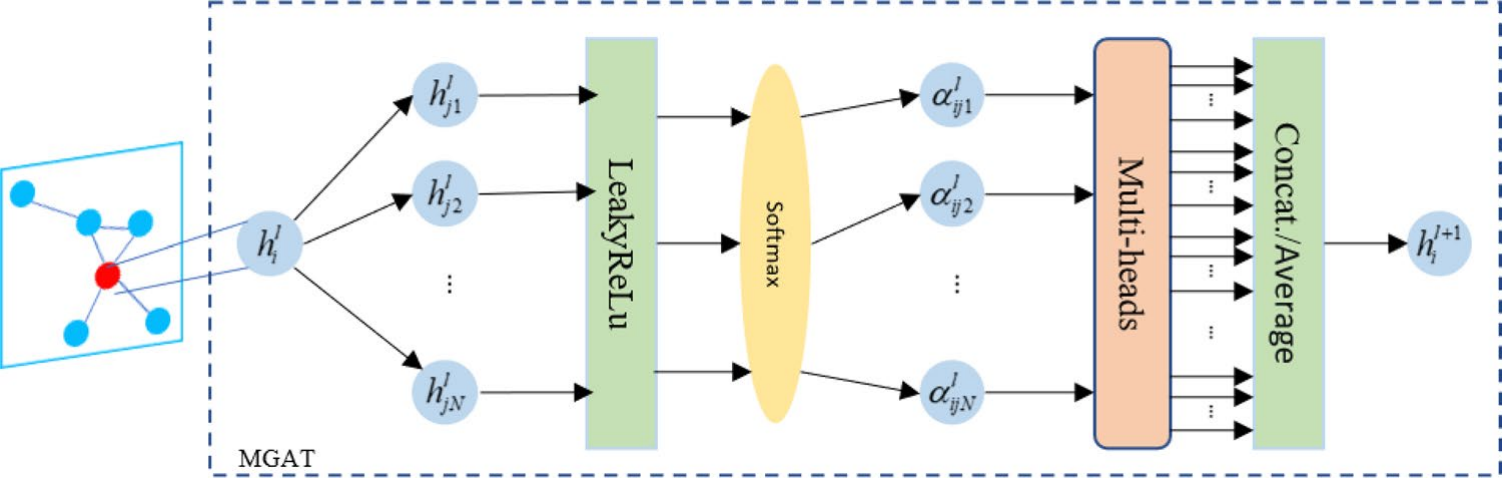
MGAT model structure.

In previous studies, the spatial dependence relationship is usually assumed to be constant, that is, the road topology is calculated only once, and the dynamic change process of traffic flow is ignored^27^. Graph Attention Network(GAT)^28^ is a spatial domain convolutional network, which uses a self-attention mechanism to learn neighbors' relative weights and aggregate neighbors' spatial features. The attention weights change with the change of data, adding dynamic spatial correlation to the network. The model structure is shown in Fig. 2. Input feature $h_{i}^{l}$ calculates the attention value of adjacent nodes according to the node attribute features, as shown in Eq. (4); After calculating the attention value of all adjacent nodes of node *i*, the *softmax* function is used to normalize the attention weight, as shown in Eq. (5); Finally, the output feature $h_{i}^{l + 1}$ is shown in Eq. (6) by aggregated attention value of neighbors.

4 $$e_{ij}^{l} = LeakyReLU\left( {a^{{l^{T} }} \left( {W^{l} h_{i}^{l} ||W^{l} h_{j}^{l} } \right)} \right)$$

5 $$\alpha_{ij}^{l} = softmax(e_{ij}^{l} ) = \frac{{\exp (e_{ij}^{l} )}}{{\sum\nolimits_{{k \in {\rm N}_{i} }} {\exp (e_{ik}^{l} )} }}$$

6 $$h_{i}^{l + 1} = \sigma \left( {\sum\limits_{{j \in {\rm N}_{i} }} {\alpha_{ij}^{l} W^{l} h_{j}^{l} } } \right)$$

where $e_{ij}^{l}$ represents the attention score on node* j i* of layer *l*; $a^{l}$ represents the learnable weight vector of layer *l*; W represents the weight matrix; $N_{i}$ represents the set of neighbor nodes of node *i*. $\sigma$ is a nonlinear activation function.

To stabilize the self-attention learning process and enhance the learning ability, the multi-head attention mechanism is used to concatenate the calculation results (as shown in Eq. 7) or average them (as shown in Eq. 8). To better capture spatial correlation, we superimposed two layers of Multi-layer Graph Attention Network (MGAT). For each time step t, flow $Q_{t} = \{ Q_{t,1} ,Q_{t,2} , \cdots ,Q_{t,N} \} ,Q_{t,i} \in R^{F}$ is input at the first layer and a new feature $h_{t}^{1} = \{ h_{t,1}^{1} ,h_{t,2}^{1} , \cdots ,h_{t,N}^{1} \} ,h_{t,i}^{1} \in R^{{F^{\prime}}}$ is output. The output features of different attention heads are spliced together as input at the second layer. The second layer outputs a new feature set $h_{t}^{2} = \{ h_{t,1}^{2} ,h_{t,2}^{2} , \cdots ,h_{t,N}^{2} \} ,h_{t,i}^{2} \in R^{{F^{\prime\prime}}}$, and averages the output features of different attention heads. To avoid gradient disappearance, the residual connection is added and the final output is $x_{t}$.

7 $$h_{i}^{l + 1} = ||_{k = 1}^{K} \sigma \left( {\sum\limits_{{j \in {\rm N}_{i} }} {\alpha_{ij}^{k} W^{k} h_{j}^{l} } } \right)$$

8 $$h_{i}^{l + 1} = \sigma \left( {\frac{1}{K}\sum\limits_{k = 1}^{K} {\sum\limits_{{j \in {\rm N}_{i} }} {\alpha_{ij}^{k} W^{k} h_{j}^{l} } } } \right)$$

where || indicates that the features are operated in series; *K* is the number of multiple attention heads; $\alpha_{ij}^{k}$ represents the attention coefficient of the *K*th attention head.

### Time feature extraction and fusion

In addition to the spatial correlation, the traffic flow shows periodic characteristics in time, that is, the traffic volume of the day is similar to the traffic flow of the previous day. Considering the influence of social fixed events such as weekly holidays on traffic flow, this paper constructs three kinds of time series $Q^{R}$、$Q^{D}$ and $Q^{W}$ along the time axis to extract features. Denote adjacent time, daily cycle time, and weekly cycle time, respectively. Assuming that the flow of the time step $t_{p}$ after $Q_{t}$ is predicted, the adjacent time series $Q^{R} = \{ Q_{t - r + 1} ,Q_{t - r + 2} , \cdots ,Q_{t} \}$ is composed of samples r steps before the current step. A daily cycle time series $Q^{D} = \{ Q_{t - d + 1} ,Q_{t - d + 2} , \cdots ,Q_{{t - d + t_{p} }} \}$ represents a sequence at the same time one day earlier. A cycle time series represents a sequence $Q^{W} = \{ Q_{t - w + 1} ,Q_{t - w + 2} , \cdots ,Q_{{t - w + t_{p} }} \}$ at the same time one week ago.

**Figure 3:**
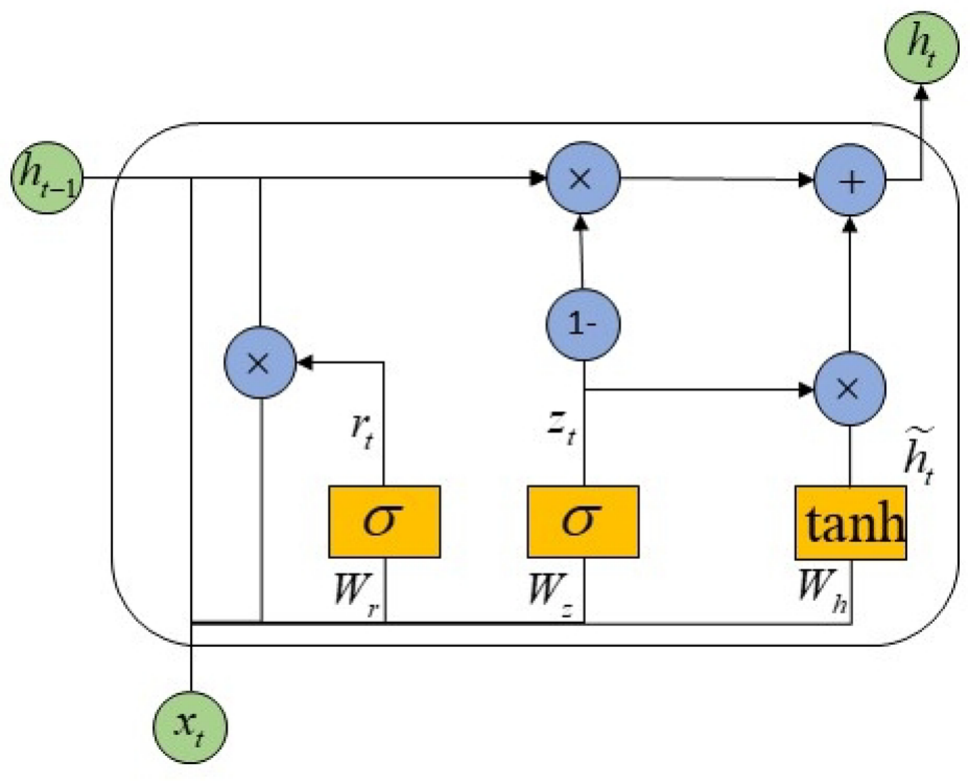
GRU structure diagram.

At present, the recurrent neural network is the most widely used in processing sequence data, but the traditional recurrent neural network has the defect of gradient explosion and gradient disappearance due to the influence of prediction length. Therefore, to solve the above problems, this paper adopts a gated recurrent unit to extract the time feature. The gated recurrent unit (GRU)^29^ is similar to the long and short-term memory Networks (LSTM) in that both use a gating mechanism to control input and memory. Compared with LSTM, GRU uses fewer parameters, has a simpler network structure, and is more efficient. LSTM has three gates, namely the input door, the forgotten door, and the output door, while GRU has only two gates, namely the updated door and the reset door. The update door is used to control how much the hidden layer state of the previous moment has been updated to the current hidden layer state. The reset gate is used to control how much the hidden layer state of the previous moment is updated to the current candidate hidden layer state. The model structure diagram is shown in Fig. 3, and the calculation process is as follows:

9 $$r_{t} = \sigma (W_{r} \cdot [h_{t - 1} ,x_{t} ] + b_{r} )$$

10 $$z_{t} = \sigma (W_{z} \cdot [h_{t - 1} ,x_{t} ] + b_{z} )$$

11 $$h_{t} = \tanh [W_{h} [r \cdot h_{t - 1} ,x_{t} ] + b_{h} ]$$

12 $$h_{t} = (1 - z_{t} ) \cdot \tilde{h}_{t} + z_{t} \cdot h_{t - 1}$$

where $r_{t}$ is the reset gate, $z_{t}$ is the update gate, $x_{t}$ is the input sample of the current moment, $h_{t - 1}$ is the hidden state of the previous moment, $\widetilde{{h_{t} }}$ is the intermediate state gate, $\sigma$ is the Sigmoid activation function, $W_{r}$,$W_{h}$,$W_{z}$ is the weight, $b_{r}$,$b_{z}$,$b_{h}$ is the bias.

Each node *i* adjacent time series, daily periodic time series, and weekly periodic time series are input into GRU to extract time features. In order to reduce the influence of redundant information on the prediction results, the adaptability of the model to the neighboring time affected by traffic incidents is increased. Therefore, the attention mechanism^30^ is introduced to capture the importance of different periodic time series data by taking the nearby time traffic flow as the main reference. The model uses the nearby time as the query matrix and the cycle time as the key matrix. The model uses the adjacent time as the query matrix and the cycle time as the key matrix. The score function of the model is shown in Eq. (13), and the attention weight is shown in Eq. (14). Finally, the attention vector $C_{t,i}^{d}$ of the daily cycle sequence is obtained, as shown in Eq. (15), and the attention vector $C_{t,i}^{w}$ of the weekly cycle sequence can be obtained similarly.

13 $$e(h_{t,i}^{r} ,h_{m,i}^{d} ) = V^{T} \tanh (W_{s,i} h_{t,i}^{r} + W_{u,i} h_{m,i}^{d} )$$

14 $$\alpha_{m,i}^{d} = sigmoid(e(h_{t,i}^{r} ,h_{m,i}^{d} )) = \frac{{\exp (e(h_{t,i}^{r} ,h_{m,i}^{d} ))}}{{\sum\nolimits_{m \in M} {\exp (e(h_{t,i}^{r} ,h_{m,i}^{d} ))} }}$$

15 $$C_{t,i}^{d} = \sum\nolimits_{m \in M} {h_{m,i}^{d} } \alpha_{m,i}^{d}$$

where *V*, $W_{s,i}$ and $W_{u,i}$ are all training parameters; $h_{t,i}^{r}$ is the neighboring time series output sequence; $h_{m,i}^{d}$ is the diurnal time series output sequence.

Finally, the adjacent time output sequence $h_{t,i}^{r}$ is fused with the daily periodic sequence attention vector $C_{t,i}^{d}$ and the weekly periodic sequence attention vector $C_{t,i}^{w}$, and the final output is obtained through fully connected layer mapping.

## Experiment

### Datasets and preprocessing

In order to verify the prediction performance of the model, PEMSD4 is used for experimental verification. The data includes 307 detection points, the data sampling time is 59 days from January to March 2018, the data sampling interval is 5 min, and each road contains 16,992 traffic flow data. The data mainly consists of three traffic parameters, including flow, average speed, and average density. We treat 307 detection points as 307 road segments and construct the link relationship according to the road network topology. The experimental dataset is divided into training sets, validation sets, and test sets according to the 6:2:2 ratio. In order to eliminate the influence of different dimensions, the data are normalized, and the normalization formula is shown in Eq. (16).

16 $$Q_{nor} = \frac{Q - mean(Q)}{{std(Q)}}$$

where $mean(Q)$ represents the mean of sequence data and $std(Q)$ represents the standard deviation of sequence data.

### Baselines

In order to evaluate the model performance, the following models are selected for comparative analysis.

HA^31^: Historical traffic data were used and the mean value was taken for prediction.

ARIMA^32^: The time series fitting parameter model is used for traffic prediction, which only needs endogenous variables and is more sensitive to capturing linear relationships.

SVR^33^: By mining the nonlinear relationship in the historical data to achieve the purpose of prediction, the kernel function can be used to map the high-dimensional data.

GRU^34^: This model is a variant of LSTM with a simpler structure and fewer parameters to reduce the risk of overfitting.

STGCN^35^: Traffic flow prediction by constructing spatiotemporal blocks. The road network topology structure was used to construct a graph to extract spatial features, and the convolutional neural network was used to extract temporal features to complete traffic prediction.

ASTGCN^24^: It considers the periodic characteristics of traffic by using temporal attention mechanism and spatial attention to capture the dynamic correlation in time and space, and then uses a graph convolutional network for traffic prediction.

### Experimental parameter setting

We implemented the DSTGAT model based on the pytorch framework. The prediction step $T_{p} = 12$ in this paper is to predict the traffic within 1 h. $T_{h} = 12,T_{d} = 12,T_{w} = 12$ is chosen to construct the time series of recent, daily, and weekly periods, respectively. The number of attention heads of GAT is set to 8 according to the performance on the validation set, the model is trained by Adam optimizer with a learning rate of 0.0001, the batch size is set to 64, dropout^36^ (dropout rate = 0.5) is used to prevent the model from overfitting, and L2Loss is used as the loss function of the model. Other baseline model parameters are shown in Table 1.

**Table 1:** Baseline model parameters.

Model	Parameters	Values	Description
HA	Time step	12	Length of the sample
ARIMA	p	5	Autoregressive order
	Time step	12	Length of the sample
	d	1	Degree of difference
	q	6	Moving average order
SVR	Time step	12	Length of the sample
	Kernel function	RBF	Data is mapped to higher dimensions
	Kernel function coefficient	200	The data is mapped to the new feature space distribution
	Coefficient of penalty	0.1	Error tolerance
GRU	Time step	12	Length of the sample
	Learning rate	0.01	Decline rate of the cost function
	Batch size	32	Number of samples in one training
STGCN	Time step	12	Length of the sample
	Learning rate	0.01	Decline rate of the cost function
	Batch size	32	Number of samples in one training
ASTGCN	Time step	12	The recent, daily and weekly time steps are all 12
	Learning rate	0.0001	Decline rate of the cost function
	Batch size	64	Number of samples in one training
	Convolution kernel	64	All the graph convolution layers use 64 convolution kernels
	K	3	The number of terms of the Chebyshev polynomial

### Evaluation metrics

In this paper, Mean Absolute Error (MAE), Root Mean Square Error (RMSE), and Mean Absolute Percentage error (MAPE) are selected to evaluate the effectiveness of the model. The calculation process is as follows:

17 $$MAE = \frac{1}{m}\sum\limits_{i = 1}^{m} {|Y_{t} - Y_{t} } |$$

18 $${\text{RMSE}} = \sqrt {\frac{1}{m}\sum\limits_{i = 1}^{m} {(Y_{t} - Y_{t} )^{2} } }$$

19 $${\text{MAPE}} = \frac{1}{m}\sum\limits_{i = 1}^{m} {\frac{{|Y_{t} - Y_{t} |}}{{Y_{t} }}}$$

where *m* represents the number of nodes in the graph *G*;$Y_{t}$ is the actual value; $\widehat{{Y_{t} }}$ is the predicted value.

### Result analysis

The proposed model and the baseline model are used to predict the traffic of the next hour on the data set, and the performance pairs are shown in Table 2. It can be seen from the table that the proposed model outperforms the baseline model in all three-evaluation metrics. Both statistical models and time series models have poor prediction performance compared with deep learning models. The reason is that for the statistical model and time series model, only the road section itself is considered, and the flow change characteristics of the road section near the target section are not considered, so the random fluctuation characteristics of the road section traffic model cannot respond well, and then the model performance is low and the model benefit is not high. For the deep learning network, compared with STGCN and ASTGCN, which perform better in the baseline model, the proposed model performs better in MAE, RMSE, and MAPE, indicating that considering the time series correlation of non-adjacent road segments and considering spatiotemporal attention features plays an important role in improving the accuracy of the model.

**Table 2:** Performance comparison of different models.

Model	RMSE	MAE	MAPE (%)
HA	57.16	39.68	32.69
ARIMA	63.31	37.05	35.34
SVR	55.87	35.36	30.26
GRU	48.13	33.17	24.27
STGCN	41.41	30.28	18.92
ASTGCN	37.23	27.32	17.58
DSTGAT	35.35	25.54	16.67

**Figure 4:**
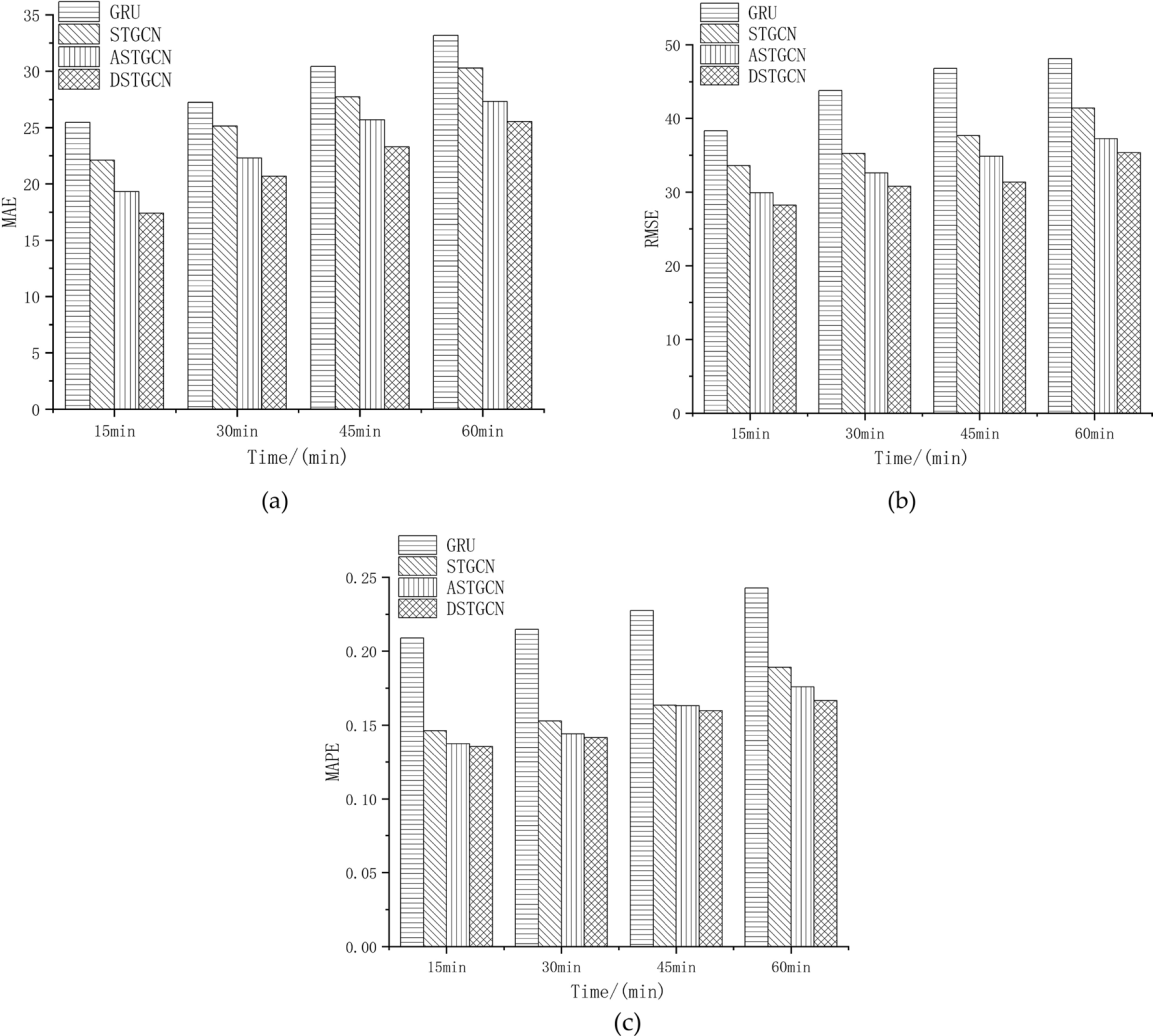
Performance comparison of different prediction step models: (**a**) MAE; (**b**) RMSE; (**c**) MAPE.

In order to verify the prediction performance of the proposed model at different time lengths, four models of the baseline model, GRU, STGCN, ASTGCN and the proposed model, are selected to predict the traffic flow in the next 15 min, 30 min, 45 min and 60 min, and the results are shown in Fig. 4. It can be seen from the figure that the three indicators of the proposed model are better than the other three baseline models under different time steps, and the prediction accuracy of all models decreases with the increase of time step. The shorter the step, the smaller the prediction error and the higher the accuracy.

We verified the impact of the proposed model components on the prediction accuracy, and the results are shown in Table 3. We used adjacency matrix (*A*) of adjacent road segments on the original model, and considered adjacency matrix (*Ap*) of non-adjacent road segments at the same time, added attention mechanism (TA) in capturing temporal correlation features, and determined Pearson coefficient *p* = 0.9 for indirect correlation features. As can be seen from the table, each component of the model contributes to the model performance. Among them, the introduction of the attention mechanism reduces the mean absolute error of the model by 7.56% and the mean absolute percentage error by 8.56%, indicating that this component has a significant effect on improving the accuracy of the model. The choice of correlation coefficient shows that the model does not improve the prediction accuracy with higher correlation coefficient. Of course, the model will not improve the efficiency of the model because of the small correlation coefficient and the inclusion of more data node information.

**Table 3:** Comparison of ablation experimental performance.

Model parameters	MAE	RMSE	MAPE (%)
*Ap*, TA, *p* = 0.9	25.54	35.35	16.67
*A*, TA	26.35	35.46	16.78
*Ap*, *p* = 0.9	27.63	36.16	18.23
*Ap*, TA, *p* = 0.80	26.11	35.45	16.73
*Ap*, TA, *p* = 0.85	25.36	35.19	16.21

## Discussion and conclusions

Aiming at the problem of traffic flow prediction, a dynamic spatiotemporal graph attention network traffic flow prediction model based on the attention mechanism was proposed. PeMSD4 was used for experimental verification and comparison analysis with multiple baseline models, and the following conclusions were obtained:

1. The upstream and downstream traffic flow has an important influence on the road section and has a certain correlation, but not all adjacent road sections are related to the traffic flow of the target road section, and affected by geographical location or other factors, non-adjacent road sections are not necessarily unrelated to the target road section. Therefore, this paper uses the Pearson correlation coefficient to evaluate the similarity of traffic flow series under a certain time granularity and constructs the adjacency matrix accordingly. Ablation experimental results show that discussing the indirect correlation of non-adjacent sections has a certain effect on improving the accuracy of the model.
2. The traffic flow has periodic characteristics, that is, the macroscopic fluctuation of traffic flow is not large, but due to the influence of microcosmic traffic events, the short-term traffic flow is extremely vulnerable to being affected and fluctuated, so the features extracted by periodic historical data cannot adapt to the fluctuation of current traffic flow caused by traffic events. Therefore, this paper introduces the attention mechanism to assign weights to periodic data concerning the current neighboring time. Experiments show that micro fluctuations have an important impact on the prediction effect.
3. The prediction accuracy of the model is affected by the prediction step size, the longer the prediction step size is, the lower the accuracy is. This paper uses the periodic characteristics of traffic flow modeling and combines the GRU model based on historical periodic data, which can better capture the long-term dependence characteristics of traffic flow. The experimental results show that the model has better benefits in the medium-long step size prediction.
4. The experimental results show that according to the traffic flow data in the near time, the spatiotemporal similarity link is constructed, the daily cycle time and weekly cycle time traffic data are combined, the influence of traffic events in the near time on traffic flow is fully considered, and the evaluation effect of the model is enhanced.

Based on the existing research results, in the future, we plan to enrich and expand the traffic flow prediction framework by integrating multi-source external data, such as weather^37^, holidays^38^, and regional POI features^39^, to enhance the capture ability of geospatial semantic information of the model. At the same time, new arriving data are processed in real-time, and a more effective prediction system is explored in the field of dynamic training^40^, to further optimize the design of the traffic flow prediction model and improve its generalization ability.

## Data Availability

Data are available from the corresponding author upon request.
